# Association of Mitochondrial DNA Copy Number and Telomere Length with Prevalent and Incident Cancer and Cancer Mortality in Women: A Prospective Swedish Population-Based Study

**DOI:** 10.3390/cancers13153842

**Published:** 2021-07-30

**Authors:** Yanni Li, Kristina Sundquist, Xiao Wang, Naiqi Zhang, Anna Hedelius, Jan Sundquist, Ashfaque A. Memon

**Affiliations:** 1 Center for Primary Health Care Research, Lund University, 20502 Malmö, Sweden; kristina.sundquist@med.lu.se (K.S.); xiao.wang@med.lu.se (X.W.); naiqi.zhang@med.lu.se (N.Z.); anna.hedelius@med.lu.se (A.H.); jan.sundquist@med.lu.se (J.S.); ashfaque.memon@med.lu.se (A.A.M.); 2 Department of Family Medicine and Community Health, Department of Population Health Science and Policy, Icahn School of Medicine at Mount Sinai, New York, NY 10029, USA; 3 Center for Community-Based Healthcare Research and Education (CoHRE), Department of Functional Pathology, School of Medicine, Shimane University, Izumo, Shimane 693-8501, Japan

**Keywords:** mitochondrial DNA copy number, relative telomere length, cancer types, prevalent cancer, cancer risk, mortality

## Abstract

**Simple Summary:**

Individuals with abnormal alterations in mitochondrial DNA copy number (mtDNA-CN) and telomere length are at higher risk of developing certain types of cancer. This report suggests that mtDNA-CN and relative telomere length measured in peripheral blood have potential clinical applications for risk prediction of different cancers and that mtDNA-CN could be used as a prognostic biomarker in malignancy. This comprehensive work strengthens several previous relevant findings in certain types of cancer and broadens our understanding of the link between mtDNA-CN, telomere length and future risk of many cancer types. The translational implication of our findings is that postmenopausal genital organ cancer patients with lower levels of baseline mtDNA-CN or shorter telomere length can be identified for early adjustment of lifestyle and hormone replacement therapy.

**Abstract:**

Changes in mitochondrial DNA copy number (mtDNA-CN) and telomere length have, separately, been proposed as risk factors for various cancer types. However, those results are conflicting. Here, mtDNA-CN and relative telomere length were measured in 3225 middle-aged women included in a large population-based prospective cohort. The baseline mtDNA-CN in patients with prevalent breast cancer was significantly higher (12.39 copies/µL) than cancer-free individuals. During an average of 15.2 years of follow-up, 520 patients were diagnosed with cancer. Lower mtDNA-CN was associated with decreased risk of genital organ cancer (hazard ratio (HR), 0.84), and shorter telomere length was associated with increased risk of urinary system cancer (HR, 1.79). Furthermore, mtDNA-CN was inversely associated with all-cause (HR, 1.20) and cancer-specific mortality (HR, 1.21) when considering all cancer types. Surprisingly, shorter telomere length was associated with decreased risk of cancer-specific mortality when considering all cancer types (HR, 0.85). Finally, lower mtDNA-CN and shorter telomere length were associated with increased risk of both all-cause and cancer-specific mortality in genital organ cancer patients. In this study population, we found that mtDNA-CN and telomere length were significantly associated with prevalent and incident cancer and cancer mortality. However, these associations were cancer type specific and need further investigation.

## 1. Introduction

Cancer is expected to rank as the leading cause of death (age < 70) worldwide and is the single most important public health problem that lacks a global solution [1]. Studies from epidemiological profiles of cancer have shown that different exposures to risk factors, lifestyles, economic settings and access to care or screening programs, for a person who develops cancer, may vary heterogeneously [2,3,4]. Nevertheless, morbidity and mortality caused by cancer in every world region pose a huge threat to global development and lay a tremendous burden on our society.

The dysfunction of mitochondria is one of the hallmarks of cancer. Mitochondria have their own genome (mtDNA, 16,596 base pair) and, according to the energy demands, their copy numbers range from a few hundred to more than 10,000 in a cell type- and origin-specific manner [5,6]. MtDNA is circular double-stranded DNA, located in the mitochondrial inner membrane close to the site where excessive reactive oxygen species (ROS) are routinely generated, and it is prone to be injured by oxidative attack [7,8,9]. The damaged mtDNA molecules are primarily resolved via robust base excision repair. However, unlike nuclear DNA, mtDNA with double-strand breaks (DSBs) is degraded rapidly instead of being repaired, leading to a significant decrease in mtDNA copy number (mtDNA-CN) [10]. Non-cleaved mtDNA is subsequently replicated by a mitochondrial replisome comprising DNA polymerase gamma (Pol γ), twinkle helicase and single-strand binding protein SSBP1 as a feedback mechanism to compensate for the metabolic defects in impaired mitochondria [11,12]. Thus, mtDNA-CN is a relative measurement that reflects mitochondrial pathologies and it is prone to alteration under various energy requirements and physiological and environmental conditions [13,14]. Nevertheless, erroneous replication and repair can contribute to accumulating mtDNA mutations, leading to mitochondrial dysfunction and signaling to the nucleus [12,15]. As an indirect biomarker for mitochondrial function, mtDNA-CN has been widely associated with many diseases, including cancer [16], aging [17,18,19], depression [20,21], cardiovascular disease [22,23], type 2 diabetes [24,25], liver disease [26,27], chronic kidney disease [28,29] and neurodegenerative disease [30]. However, current studies on the mtDNA-CN in cancer have reported mixed results; most were based on a case–control design and were inconsistent for various types of cancers.

Telomeres are the nucleoprotein complexes crucial in preserving chromosomal stability and integrity; their length ranges from 5 to 15 kb in humans and varies among tissues [31]. Telomerase is the enzyme responsible for maintaining telomere length and is silenced in normal somatic cells. In the absence of maintenance mechanisms, telomeres undergo shortening with cell division in most human tissues, reflecting organism aging at the cellular level influenced by oxidative stress [32,33,34,35]. Short telomeres eventually trigger cellular senescence and a DNA damage signal where cells will stay in a quiescent state for years and secrete factors that influence aging-related diseases rather than undergo apoptosis, which was suggested as a tumor suppressor mechanism for humans [36]. However, the abnormal or extreme shortening of telomere length may cause chromosomal degradation and contribute to malignant cell transformation, which is associated with a higher risk of multiple human diseases, including cancer [37,38]. Telomere shortening has a dual role in carcinogenesis. It promotes the initiation of cancer by inducing chromosomal instability, while telomere length maintenance characterized by telomerase expression is required for cancer cell proliferation and tumor growth [39]. Similar to mtDNA-CN, the reports on telomere length as a biomarker for cancer risk are contradictory.

MtDNA and telomere length are highly variable across cell types but maintained within a constant range according to the specific tissue, therefore, mtDNA-CN and telomere length measured in peripheral blood are considered a surrogate for the measurement of personal health outcomes. Both mitochondria and telomeres serve as critical regulators of the aging process, and their structures are easily damaged by ROS and systemic inflammation; they also play important roles in tumorigenesis [40,41,42]. The conclusions drawn from previous studies showed conflicting results on the associations between mtDNA-CN or telomere length and risk of cancers. Possible explanations could be sample collection, sample selection, study design and measurement errors. Few prospective studies have been performed on telomere length and cancer risk. However, to the best of our knowledge, no prospective study has been conducted on mtDNA-CN and all cancer incidence. Furthermore, the most popular techniques for quantification of mtDNA-CN and telomere length are PCR based, which in most cases provide relative measurements. Moreover, there is no study available with a focus on population-based studies systematically analyzing the association between mtDNA-CN, telomere length and the prevalence, incidence and mortality of all cancer types. We aimed to comprehensively explore this possibility in a large cohort of middle-aged Swedish women with precisely quantified mtDNA-CN and telomere length from our well-optimized droplet digital (dd) PCR and quantitative real-time (qRT) PCR methods, respectively. We hypothesized that mtDNA-CN and telomere length are potential biomarkers for the identification of prevalent cancers as well as for the prediction of incident cancers.

## 2. Materials and Methods

### 2.1. Study Population

The present study was conducted based on Women’s Health in Lund Area (WHILA), a prospective population-based cohort that started in 1995. All women, aged 50–59 years (born between 1935 and 1945) and living in Scania in southern Sweden, were invited to participate in a health survey. From December 1995 to February 2000, a total of 6917 women (out of 10,766, the total population of women in the five southern municipalities in 1995) underwent a physical examination and answered a questionnaire. There was no financial reimbursement for participation. After providing written consent, the participants were given up to two hours to answer the questionnaire. The questionnaire that was distributed to all participants has been described previously [43]. If they had any uncertainties, they could ask an experienced research nurse for assistance. Participants were followed from the day of screening until death, or if no event occurred, until 31 May 2015. However, the blood samples for DNA extraction were collected midway through this study (from October 1997) and therefore 3225 participants were included in the present study.

### 2.2. Outcome Measurement

Information about cancer incidence and mortality was obtained from the Swedish Cancer Registry and Death Registry and information on prevalent cancer was obtained from self-reported data from questionnaires. Among the participants included in the study, 187 individuals were diagnosed with cancer at baseline (prevalent cancer) and 3038 individuals were cancer-free at baseline. We followed the cancer-free women from the day of screening until (1) cancer diagnosis; (2) death; (3) ending date of this study (31 May 2015). Individuals’ diagnoses of cancer were then identified and followed until death from any cause (overall mortality) and from cancer (cancer mortality) and/or till the end of the study period, whichever came first.

The following cancer outcomes were classified according to the WHO’s International Classification of Diseases (revision 10) as (a) breast cancer; (b) digestive system cancer (liver cancer, pancreatic cancer, gastric cancer, small intestine cancer, rectum cancer, colon cancer and oral cancer); (c) respiratory system cancer (lung cancer); (d) genital organ cancer (ovary cancer, cervix cancer, uterus cancer and corpus cancer); (e) urinary system cancer (kidney cancer and urethral cancer); (f) hematological cancer (myeloma, leukemia and non-Hodgkin’s lymphoma); (g) nervous system cancer; (h) melanoma and other malignant neoplasms of the skin; (i) endocrine gland cancer (thyroid cancer).

### 2.3. Extraction of DNA

Peripheral blood samples were collected in ethylenediaminetetraacetic acid (EDTA) tubes. Total genomic DNA was extracted using a QiAamp96 DNA Blood (Qiagen, Inc., Hilden, Germany) from a 200 μL blood sample according to the manufacturer’s instructions. The concentrations and purities of isolated DNA samples were spectrometrically analyzed and frozen at −20 °C for further usage.

### 2.4. Quantification of Relative Telomere Length

Genomic DNA extracted from blood was quantified by a Nanodrop (ND-2000, Thermo Scientific, Waltham, MA, USA) and then normalized to 5 ng/uL in TE buffer that contained Escherichia coli DNA. The DNA was heated at 95 °C for 30 min, then followed by 1 min on ice, spun down briefly at 1000× *g* at 4 °C and kept at 4 °C. Telomere length was measured by real-time PCR based on a previous report by Cawthon [44] and modified by our group.

The copy number of telomeric repeats was compared to a single copy gene (β-hemoglobin, HBG) to normalize the quantity of the input DNA. The telomere to HBG (T/S) ratio represents the average relative length of the telomeres. Detailed methods have been described previously [45]. Briefly, 20 ng DNA from samples and 7 references (from Jurkat cell line) were pooled in triplicate in 384-well plates, qPCR was performed separately for telomeres and HBG and negative controls were included. As for the measurement of telomere length, a standard curve from reference DNA was generated (Bio-Rad CFX Manager software v. 2.0.) and used in each assay plate. Telomere and HBG concentrations were calculated according to the standard curve. All standard curves for both telomere and HBG had correlation coefficients of R^2^ > 0.99. The PCR efficiencies for each reaction were higher than 93%. The inter- and intracoefficients of variation (CV) for the T/S ratios were 6.2% and 3%, respectively.

### 2.5. Quantification of MtDNA Copy Number

Droplet digital PCR (ddPCR) was used to quantify the absolute copy number of nuclear DNA (nDNA) and mtDNA. The mtDNA/nDNA content was assessed using specific primers designed to target the mitochondrial MT-ND1 (assay ID: dHsaCPE5029120) gene and nuclear EIF2C1 (assay ID: dHsaCP1000002) gene. Probes targeting nDNA were attached to a HEX fluorophore whereas mtDNA was attached to FAM and had an Iowa Black^®^ FQ quencher on all probes. All primer and probes were obtained from Bio-Rad (Hercules, CA, USA). Quality control for every step of our well-optimized ddPCR method was stringent, as described previously [46]. Briefly, 1ng DNA from samples, including positive and negative controls, was separately pooled in a 20 uL multiplex reaction containing primers (900 nM), probes (250 nM), ddPCR Supermix for probes (no UTP, 2X) and 5U/reaction restriction enzyme (HindIII). The plate with reactions was sealed and incubated at room temperature for 20 min to allow restriction enzyme digestion and then loaded into the automated droplet generator to generate droplets, followed by end-point PCR. The after-PCR plate was kept overnight at 4 °C to maximize the droplet recovery. The plate was finally read on the droplet reader, and data were collected and analyzed using QuantaSoft™ Software to calculate the numbers of positive and negative droplets in each sample. The fraction of positive droplets was then fitted to a Poisson distribution to determine the absolute copy number in units of copies/µL. The inter- and intra-CVs for absolute quantification of mtDNA-CN were 4.2% and 3.1%, respectively.

### 2.6. Assessment of Covariates

We collected information on potential confounding factors at baseline through the health survey, including age at screening, body mass index (BMI), education (1–9, 10–11, ≥12 years of schooling), alcohol habits (no consumption, <12 g/day, ≥12 g/day) and smoking habits as non-smokers, past smokers (≥1 pack year, stopped smoking ≥1 month prior to the study) and current smokers (≥1 pack year). Physical activity at home was defined according to the questionnaire and the participants with a score of 1–3 were categorized as low activity at home: 1 = hardly do anything at all, 2 = mostly sedentary, 3 = light physical exertion. High activity at home was categorized with a score of 4–6: 4 = strenuous exercise 1–2 h/week, 5 = strenuous exercise at least 3 h/week, 6 = hard regular exercise. Physical activity at work was categorized as low, moderate and high. Information on comorbidity was collected from both baseline self-reported questionnaires and the Swedish health registries concerning diabetes (including type 1 and type 2 diabetes as yes/no), cardiovascular disease (CVD, including stroke, coronary heart disease, abdominal aortic aneurysm as yes/no) and hypertension (yes/no).

### 2.7. Statistics

A Pearson chi-square test was used to compare categorical variables (education level, smoking habits, alcohol consumption, activity at work, activity at home, diabetes, CVD, hypertension) and continuous variables (age at screening, BMI) were compared using Student’s *t*-tests. Linear regression analysis was performed to evaluate the association between prevalent cancer (yes/no) and mtDNA-CN or telomere length at baseline. We further produced a Cox proportional hazards model to explore the association between mtDNA-CN, telomere length and cancer incidence in 3038 cancer-free individuals. Subjects were dichotomized into high and low mtDNA or long and short telomere length groups according to the median based on the distribution of mtDNA-CN or telomere length. The high or long group served as the reference group in the analyses. Hazard ratios (HRs) and 95% confidence intervals (95% CIs) were calculated to evaluate the association between mtDNA-CN, telomere length and cancer risk. We further examined the association between mtDNA-CN, telomere length and all-cause mortality, as well as cancer-specific mortality in 520 cancer patients. Competing risk models were created while analyzing cancer-specific mortality. Deaths from other causes were considered as competing risks. Kaplan–Meier survival curves were calculated to evaluate the association between mtDNA-CN and telomere length and cancer mortality. To control for potential confounders, the following variables were included in the multivariate regression model: age, BMI, education level, smoking habits, alcohol consumption, activity at work, activity at home, diabetes, CVD and hypertension. All statistical analyses were carried out in SPSS software version 23 (IBM, Armonk, NY, USA) and SAS version 9.4. 

## 3. Results

**Figure 1:**
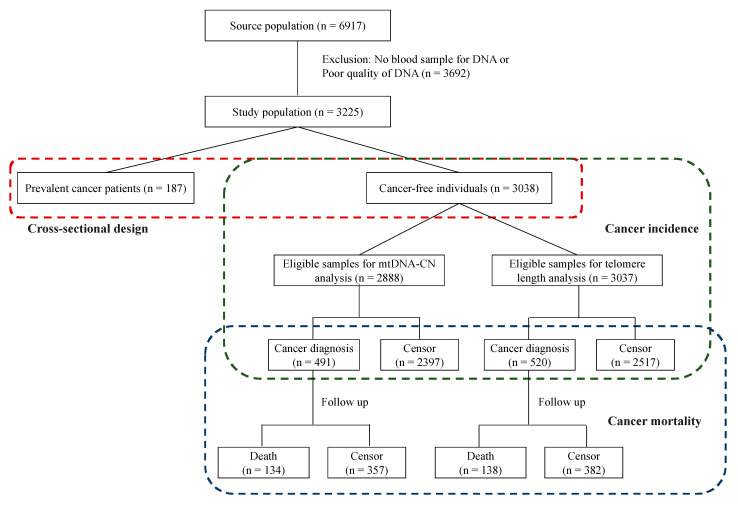
Flow chart of participants in this cohort study.

Of the 3225 participants who had their blood samples collected at baseline and were included in this study, 187 (5.8%) were reported as having prevalent cancer and 3038 women without cancer were followed for incident cancer. During an average 15.2 years of follow-up, 520 of 3038 participants (17.1%) developed cancer and, among them, 138 died during the follow-up (Figure 1).

### 3.1. Population Characteristic of Prevalent Cancer and No Cancer at Baseline

Table 1 shows the characteristics of the study population at baseline. Compared to cancer-free individuals, cases were older and less likely to consume alcohol (*p* < 0.05). Telomere length (mean ± SD) was normally distributed and was shorter in participants with prevalent cancer. No significant differences were observed between cancer-free individuals and prevalent cancer patients in terms of BMI, education level, smoking habits, activity at work, activity at home, diabetes, CVD or hypertension. All of the variables referenced above were considered as potential confounders and were adjusted in the subsequent multivariable analyses.

### 3.2. Prevalent Cancer and MtDNA-CN/Telomere Length

We performed further crude and adjusted linear regression analysis to investigate the association between prevalent cancer and mtDNA-CN or telomere length. The cancer diagnoses were categorized into cancer types according to ICD codes to determine whether the results applied to site-specific cancers. All cancers in this study were categorized across the nine main cancer types in the following way: breast, digestive system, respiratory system, genital organ, urinary system, hematological tumor, nervous system, skin and endocrine gland cancer (Table S1).

Our results show that prevalent breast cancer was significantly associated with higher mtDNA-CN (adjusted β was 12.39; 95% CI = 4.15, 20.63; *p* = 0.003). An inverse association between prevalent hematological cancer and mtDNA-CN was found, however, it did not reach statistical significance (adjusted β was −25.21; 95% CI = −51.19, 0.77; *p* = 0.057) (Table 2).

Furthermore, prevalent cancer was significantly associated with shorter telomere length (crude β was −0.03; 95% CI = −0.05, −0.01; *p* = 0.027). However, this association became non-significant after adjusting for potential confounders (adjusted β was −0.02; 95% CI = −0.04, 0; *p* = 0.059). Stratification of the data according to the cancer types suggested that the associations between telomere length and breast and genital cancers were stronger; however, the results did not reach statistical significance (Table 3).

### 3.3. Cancer Incidences and MtDNA-CN/Telomere Length

Baseline characteristics of participants with no cancer at baseline are shown in Table S2. MtDNA-CN and telomere length were normally distributed. A significant decrease in mtDNA-CN and telomere length was seen with age (*p* < 0.001, Figures S1 and S2). For mtDNA-CN, further associations were observed for the following variables: education level, smoking habits, alcohol habits, activity at work, diabetes, CVD. Baseline telomere length was shorter in participants with higher BMI and less physical activity (Table S2).

During an average of 15.2 years of follow-up, we identified 520 patients with a cancer diagnosis. To determine if the level of mtDNA-CN or telomere length was associated with cancer risk, single-factor Cox regression analyses were conducted (Table 4). Participants with a lower level of mtDNA-CN at baseline had a lower risk of having genital organ cancer during follow-up, and the hazard ratio (HR) per one standard deviation (SD) decrease in mtDNA-CN for incident genital organ cancer was 0.84 (95% CI = 0.72, 0.98). Individuals with lower mtDNA-CN had increased risks of developing urinary system cancer (adjusted HR 8.2, 95% CI, 1.06–63.2) and hematological cancer (adjusted HR 1.97, 95% CI, 1.02–3.81). No other cancer type was significantly associated with mtDNA-CN. For a 1 SD decrease in the telomere length, the risk for incident urinary system cancer increased 1.79 times (adjusted HR 1.79, 95% CI = 1.05, 3.07). The results showed a similar trend when dichotomizing the mtDNA-CN (low, ≤111 copies/µL; high, >111 copies/µL) and telomere length (short, ≤0.721965; long, >0.721965) according to the median into two groups. Furthermore, the interactions for mtDNA-CN and telomere length for urinary system cancer and hematological cancer were statistically significant.

### 3.4. Mortality and MtDNA-CN/Telomere Length

During the follow-up of 520 cancer patients, a total of 138 participants died (all-cause mortality), and we also investigated the association between cancer mortality and mtDNA-CN or telomere length. The Kaplan–Meier plots are presented in Supplementary Figures S3 and S4.

We found that lower mtDNA-CN at baseline was associated with increased all-cause mortality (multivariable HR per 1 SD decrease, 1.20; 95% CI = 1.01, 1.42) as well as cancer-specific mortality when considering all cancer types (multivariable HR per 1 SD decrease, 1.21; 95% CI = 1.01, 1.45). Stratification of data, according to cancer type, showed an association between mtDNA-CN and all-cause mortality and cancer-specific mortality in genital cancer patients. The risk for all-cause mortality increased 2.15 times (adjusted HR 2.15, 95% CI = 1.04, 4.44) and cancer-specific mortality increased 2.42 times (adjusted HR 2.42, 95% CI = 1.03, 5.70) for a 1 SD decrease in mtDNA-CN after adjusting for potential confounders. We also dichotomized mtDNA-CN levels according to the median and our results showed that compared with participants in the higher mtDNA-CN group, the multivariable HR for mortality from all causes in genital cancer patients was 8.06 (95% CI = 1.75, 37.2) and mortality from specific genital cancer was 5.59 (95% CI = 1.61, 19.4) in the lower level mtDNA-CN group (Table 5).

The HR per 1 SD decrease in telomere length for cancer-specific mortality was 0.85 (95% CI, 0.21–1.00, multivariable model) when considering all cancer types. Similar to mtDNA-CN, shorter telomere length at baseline was associated with increased risk of all-cause mortality (adjusted HR per 1 SD decrease was 2.23; 95% CI = 1.00, 4.52) and cancer-specific mortality (adjusted HR per 1 SD decrease was 1.98; 95% CI = 1.10, 3.53) in genital cancer patients. Of note, both a lower level of mtDNA-CN and shorter telomere length were preferentially associated with increased mortality in patients with genital cancer such as ovary, cervix, uterus and corpus cancer.

## 4. Discussion

To the best of our knowledge, this prospective cohort study is the first population-based study to comprehensively explore the association between mtDNA-CN and telomere length and cancer prevalence and incidence, as well as cancer mortality, among middle-aged women. Our results show that both mtDNA-CN and telomere length are associated with the prevalence as well as with future risk of cancer but in a cancer-specific manner. Our results also show that mtDNA-CN was inversely associated with all-cause mortality and cancer-specific mortality when considering all cancer types. Finally, shorter telomere length was associated with a lower risk of cancer-specific mortality in all cancer types and breast cancer. However, in genital cancer, lower mtDNA-CN and shorter telomere length were associated with increased risk of all-cause mortality and cancer-specific mortality.

### 4.1. Comparison with Previous Studies

#### 4.1.1. MtDNA-CN, Relative Telomere Length and Prevalent Cancer

Although we found that there was no significant association between mtDNA-CN and overall prevalent cancer, we demonstrated that patients with prevalent breast cancer had higher mtDNA-CN compared with cancer-free individuals. Consistent with our result, a meta-analysis including 21 prospective studies and 17 retrospective case–control studies also suggested no significant association between mtDNA-CN and overall prevalent cancer [47]. Together, these results suggest that the association between higher mtDNA-CN and prevalent cancer may be study population and cancer type specific and this could be one of the reasons for the conflicting results published to date.

We found that prevalent cancer was associated with shorter telomere length, but this association decreased after adjusting for potential confounders. In agreement with our result, a meta-analysis of 46 retrospective observational studies also demonstrated a borderline significant relationship between telomere length and overall prevalent cancer [48].

#### 4.1.2. Baseline Levels of MtDNA-CN, Relative Telomere Length and Cancer Incidence

Our results demonstrated that a lower level of baseline mtDNA-CN was associated with a lower future risk of genital organ cancer, urinary system cancer and hematological cancer. Thus far, few prospective studies have been performed to investigate the association between baseline mtDNA-CN and future cancer risk. Consistent with our results, a nested case–control study observed a positive association between mtDNA-CN and risk in renal cells [49]. Furthermore, two prospective studies also supported the hypothesis that higher mtDNA-CN was associated with increased risk of chronic lymphocytic leukemia/small lymphocytic lymphoma [50] and non-Hodgkin’s lymphoma [51].

We observed that women with shorter telomere length had a higher risk of urinary system cancer. However, the result from another prospective study did not support a significant association between leukocyte telomere length and future risk of renal cell carcinoma [52].

#### 4.1.3. Baseline MtDNA-CN, Relative Telomere Length and Mortality in Cancer Patients

Our results show that lower baseline mtDNA-CN was associated with increased all-cause mortality as well as cancer-specific mortality in all cancer types, which is consistent with the result of the previous meta-analysis [53]. When categorized according to cancer type, lower baseline mtDNA-CN was associated with genital cancer mortality.

We observed heterogeneous associations between telomere length and mortality in different cancer types. Shorter baseline telomere length was associated with increased all-cause mortality as well as cancer-specific mortality in genital organ cancer, but decreased cancer-specific mortality in breast cancer. These inconsistent results across cancer types may reflect different carcinogenic mechanisms conferred by specific telomeres in specific cancer types. A previous systematic review suggested that shorter telomere length was associated with poorer outcomes, which supported our result [54]. Shanta et al. reported a significant association between shorter telomere length and poorer overall survival and progression-free survival in patients with ovary cancer and cervical cancer [55]. However, another study from Kotsopoulos et al. did not support a significant relationship in ovary cancer patients [56].

Smoking is known to be significantly associated with the risk of several cancer types such as cancer in the respiratory system, digestive system and urinary system [57]. Smoking was also inversely associated with both mtDNA-CN and telomere length [9,58]. We further investigated the association between cancer incidence and/or mortality and mtDNA-CN and/or telomere length stratified by smoking status. Our result showed that the risk of all-cause mortality increased 2.63 times (95% CI = 1.19, 5.83) and cancer-specific mortality 2.59 times (95% CI = 1.17, 5.42) for current or past smokers with low mtDNA-CN levels (Table S3).

### 4.2. Potential Biological Mechanisms

Mitochondria are essential organelles that generate energy in the form of ATP through respiration and oxidative phosphorylation (OXPHOS), produce ROS and initiate and execute apoptosis. In cancers, the malfunctioning mitochondria shift metabolism from OXPHOS to aerobic glycolysis, which has been suggested as a hallmark of carcinogenesis [59]. Mitochondrial dysfunction links to a decrease in apoptosis, an elevated level of ROS and the activation of the hypoxia-like pathway, which also affects nuclear gene expression and methylation [60,61]. MtDNA-CN, as a proxy for mitochondria function, has been shown to differ between cancer tissues and corresponding normal tissues for a number of cancer types, and its alterations in cancer appear to be tissue and tumor stage specific [62]. In addition, average mtDNA-CN levels in blood decrease after the age of 50 in healthy people [63]. However, little is known about the mechanisms that lead to the alteration in mtDNA-CN and the factors involved in the tissue-specific changes in cancers. Extensive genetic studies offer evidence that polymorphic mutations are significantly associated with mtDNA-CN levels and they seem to be context specific [64,65,66]. In addition to genetic factors, a few studies also showed that different exposures to various chemicals, risk factors, lifestyles, economic settings and health care systems significantly influence mtDNA-CN [67,68,69,70]. Thus, the changes in mtDNA-CN might directly depend on the type of mutations in nuclear DNA or mtDNA and/or be an adaptive response towards the effect of the mutations in order to gain a growth advantage for certain types of tumors [71].

Telomeres are specialized structures that protect the ends of chromosomes from fusion and DNA damage. Telomere length—a complex hereditary trait—seems to be a mitotic clock of the lifespan of the cells; its maintenance has been widely studied but is not well understood [72]. The telomerase enzyme plays a dominant role in maintaining and regulating telomere length, and is upregulated in tumors compared with normal tissue counterparts in over 90% of cancers. A subset of tumors employ a telomerase-independent, homologous recombination-based mechanism called alternative lengthening of telomeres (ALT) to elongate telomere length [73]. In cancers, a paradox about telomere length exists; individuals with long telomeres have a higher risk for the majority of cancers while cancerous tissues have short telomeres. Given that aging is the major risk factor for cancers, telomeres in somatic cells are typically shorter in older populations [74]. Short telomere length combined with other oncogenic changes might impair immune surveillance and lead to carcinogenesis [75]. Tumor cells that undergo oncogenic changes continue to divide and bypass the senescence, and this stage is accomplished by either upregulation or reactivation of telomerase expression, or by acquiring rarer ALT mechanisms to maintain these very short telomeres to achieve cell immortality [72]. GWASs and other studies conducted on different populations reported the identification of 18 multiple SNPs and rare variants that were associated with telomere length [76,77]. Some studies have also shown that carcinogen exposure, oxidative stress, inflammation, lifestyle and physiological stress were associated with telomere dynamics [76].

Mitochondrial DNA and telomeres have been implicated in the aging process for a long time. Growing evidence shows that telomere attrition regulates mitochondrial biogenesis and function through the PARP1-NAD^+^-SIRT1, ATM/R-P53-PGC1α/β and ATM-AKT-mTOR-PGC1β pathways, eventually resulting in mitochondrial dysfunction and increased ROS generation [78]. Beyond aging, studies have also revealed the importance of the telomere–p53–mitochondrial *axis* for cancer [79]. Therefore, further research is necessary to elucidate the biological mechanism underlying the telomere and mitochondrion connection.

### 4.3. Clinical Relevance

Our findings suggest that potential clinical applications of mtDNA-CN or telomere length as tools for risk prediction of different cancers and mtDNA-CN might be used as a prognostic biomarker of malignancy. For example, based on our results, for genital organ cancers, which are hormone-associated cancers, postmenopause with a lower level of baseline mtDNA-CN or shorter telomere length will be suggested as an indicator of poor health status, and therefore we can identify individuals for adjustment of lifestyle or for hormone replacement therapy. However, the conflicting associations between mtDNA-CN, telomere length and risk of cancer suggest that the application of these biomarkers to the general population may be premature at this stage.

### 4.4. Strengths and Limitations

As a whole, our study has important merits. This is a population-based study with a cohort followed prospectively for up to 20 years with a large population size. Second, we performed the analysis only on middle-aged women and therefore it is not confounded by variations in age and sex. Third, methodological bias is one of the main factors for conflicting results published to date, but here, we used our well-optimized methods for measurement of mtDNA-CN and telomere length to make the findings more consistent and reliable. Compared to real-time PCR, our well-established ddPCR method does not require external standards, has greater precision and improved reproducibility to provide a rigorous quantification of the absolute mtDNA-CN [46]. Fourth, cancer cases were defined by a questionnaire and the Swedish Cancer Register, so we had complete information on cancer diagnosis and death during long-term follow-up.

Nevertheless, there are a few limitations to our study. First, for participants with prevalent cancer, we do not have information on whether they underwent any chemotherapy or radiation therapy when the blood samples were drawn at baseline. Previous studies indicate that cancer treatment alters mtDNA-CN and telomere length [80,81,82]; thus, we cannot completely rule out the influence of treatment on the changes in mtDNA-CN and telomere length. Second, although our sample size is sufficient for the overall analysis, it is limited to specific cancer types with a small number of cases. The power was also limited for the analysis of prevalent cancer.

## 5. Conclusions

To the best of our knowledge, this is the first molecular epidemiological study in which we have simultaneously investigated the associations between mtDNA-CN, telomere length and prevalence, incidence and mortality of all cancer types in a large population-based prospective study. Our study strengthens several previous relevant findings and extends our understanding of the link between mtDNA-CN, telomere length and future risk of several cancer types. Further research is required to validate our results before the application of mtDNA-CN and telomere length as cancer biomarkers.

## Figures and Tables

**Table 1:** Baseline characteristics of mtDNA-CN and telomere length stratified by prevalent and no cancer.

Characteristics	Prevalent Cancer (*n* = 187)		No Cancer (*n* = 3038)		*p*-Value ^a^
	Mean	SD	Mean	SD	
Age	57.6	2.8	57.1	2.9	0.027
BMI	25.7	4.3	25.7	4.1	0.848
mtDNA-CN	111.7	40.8	109.0	35.1	0.385
Telomere length	0.69	0.15	0.72	0.15	0.027
	**Number**	**%**	**Number**	**%**	
Education level					0.755
0–9	112	57.1	1736	59.9	
10–11	25	14.0	424	13.4	
≥12	50	28.9	878	26.7	
Smoking habit					0.585
Non-smokers	151	79.9	2422	80.7	
Past smokers	6	1.8	55	2.7	
Current smokers	31	18.5	561	16.6	
Alcohol habit					0.048
No consumption	62	33.2	761	25.0	
<12 g/day	105	56.1	1908	62.8	
≥12 g/day	20	10.7	369	12.1	
Activity at work					0.866
Low	51	27.3	866	28.5	
Moderate	88	47.1	1441	47.4	
High	48	25.7	731	24.1	
Activity at home					0.174
Low	119	63.6	1780	58.6	
High	68	36.4	1258	41.4	
Comorbidity					
Diabetes	34	18.2	416	13.7	0.086
CVD	39	20.9	547	18.0	0.327
Hypertension	79	42.2	1266	41.7	0.877

CVD indicates cardiovascular disease. ^a^ Student’s *t*-tests were performed for continuous variables. Chi-square tests were performed for categorical variables.

**Table 2:** Linear regression models examining association between prevalent cancer and mtDNA-CN.

Characteristics	No. of Cases	Crude Univariate		Adjusted Univariate ^a^	
		Β (95% CI)	*p*-Value	β (95% CI)	*p*-Value
**Prevalent cancer**	174	2.75 (−2.67, 8.18)	0.320	4.18 (−1.18, 9.55)	0.059
Breast cancer	71	10.70 (2.36, 19.03)	0.012	12.39 (4.15, 20.63)	0.003
Digestive system	11	−6.53 (−27.52, 14.46)	0.351	−6.54 (−27.27, 14.20)	0.537
Respiratory system	1	3.86 (−65.64, 73.36)	0.913	1.27 (−67.36, 69.90)	0.971
Genital organs	30	−3.48 (−16.23, 9.27)	0.593	−0.48 (−13.09, 12.13)	0.941
Urinary system	5	−24.98 (−56.07, 6.11)	0.115	−20.77 (−51.47, 9.94)	0.185
Hematological cancer	7	−25.23 (−51.51, 1.05)	0.060	−25.21 (−51.19, 0.77)	0.057
Nervous system	5	−10.76 (−41.86, 20.34)	0.498	−8.80 (−39.53, 21.93)	0.575
Skin	17	11.27 (−5.62, 28.17)	0.191	9.51 (−7.19, 26.21)	0.264
Endocrine glands	17	−2.57 (−19.47, 14.47)	0.766	−0.88 (−17.58, 15.84)	0.918

^a^ Adjusted for age, body mass index (BMI), education level, smoking habits, alcohol consumption, activity at work, activity at home, diabetes, CVD, hypertension.

**Table 3:** Linear regression models examining association between prevalent cancer and telomere length.

Characteristics	No. of Cases	Crude Univariate		Adjusted Univariate ^a^	
		β (95% CI)	*p*-Value	β (95% CI)	*p*-Value
**Prevalent cancer**	187	−0.03 (−0.05, −0.01)	0.027	−0.02 (−0.04, 0)	0.059
Breast cancer	77	−0.03 (0.06, 0.01)	0.098	−0.03 (−0.06, 0.01)	0.143
Digestive system	11	−0.02 (−0.11, 0.07)	0.697	−0.02 (−0.11, 0.07)	0.683
Respiratory system	1	−0.08 (−0.38, 0.22)	0.592	−0.08 (−0.38, 0.22)	0.597
Genital organs	35	−0.05 (−0.10, 0)	0.069	−0.04 (−0.09, 0.01)	0.119
Urinary system	5	−0.05 (−0.18, 0.09)	0.472	−0.03 (−0.17, 0.10)	0.635
Hematological cancer	7	0.08 (−0.04, 0.19)	0.175	0.08 (−0.04, 0.19)	0.191
Nervous system	5	−0.08 (−0.21, 0.05)	0.219	−0.08 (−0.21, 0.06)	0.256
Skin	18	−0.02 (−0.09, 0.06)	0.687	−0.02 (−0.09, 0.05)	0.628
Endocrine glands	17	−0.01 (−0.09, 0.06)	0.704	−0.01 (−0.08, 0.07)	0.834

^a^ Adjusted for age, BMI, education level, smoking habits, alcohol consumption, activity at work, activity at home, diabetes, CVD, hypertension.

**Table 4:** Hazard ratios and 95% confidence intervals of cancer incidence associated with mtDNA-CN (*n* = 2888) and relative telomere length (*n* = 3037).

Characteristics	MtDNA-CN		HR (95% CI) per 1-SD Decrease in mtDNA-CN	Relative Telomere Length		HR (95% CI) per 1-SD Decrease in Telomere Length	*p* for Interaction ^c^
	High (*n* = 1406)	Low (*n* = 1482)		Long (*n* = 1473)	Short (*n* = 1564)		
**All cancer**							
No. of cancer diagnoses	242	249		256	264		
Person-years of follow-up	21,700	22,238		23,394	22,626		
IR, per 1000 person-years	11.15	11.19		10.94	11.67		
Crude HR (95% CI)	1 (Ref)	1.04 (0.87–1.24)	1.01 (0.92–1.10)	1 (Ref)	0.97 (0.82–1.16)	1.02 (0.94–1.11)	
Adjusted HR (95% CI) ^a^	1 (Ref)	0.99 (0.83–1.19)	0.99 (0.90–1.08)	1 (Ref)	0.93 (0.78–1.10)	1.00 (0.92–1.09)	0.607
**Cancer types** (adjusted HR and 95% CI ^a^)							
Breast cancer	1 (Ref)	1.05 (0.78–1.42)	0.95 (0.84–1.08)	1 (Ref)	1.03 (0.77–1.38)	0.98 (0.84–1.14)	0.197
Digestive system	1 (Ref)	0.83 (0.54–1.29)	1.03 (0.84–1.26)	1 (Ref)	1.11 (0.71–1.72)	1.05 (0.88–1.25)	0.225
Respiratory system	1 (Ref)	0.91 (0.42–2.02)	1.18 (0.88–1.60)	1 (Ref)	0.58 (0.29–1.16)	0.83 (0.63–1.10)	0.192
Genital organs	1 (Ref)	0.60 (0.33–1.10)	0.84 (0.72–0.98) ^b^	1 (Ref)	1.16 (0.66–2.05)	1.13 (0.87–1.47)	0.207
Urinary system	1 (Ref)	8.20 (1.06–63.2) ^b^	1.08 (0.72–1.63)	1 (Ref)	1.35 (0.45–4.08)	1.79 (1.05–3.07) ^b^	<0.001
Hematological cancer	1 (Ref)	1.97 (1.02–3.81) ^b^	1.11 (0.85–1.46)	1 (Ref)	0.81 (0.43–1.54)	0.97 (0.71–1.32)	0.007
Nervous system	1 (Ref)	1.62 (0.49–5.31)	1.07 (0.66–1.72)	1 (Ref)	0.68 (0.22–2.07)	0.74 (0.45–1.22)	0.314
Skin	1 (Ref)	0.82 (0.49–1.39)	1.05 (0.82–1.34)	1 (Ref)	0.81 (0.50–1.32)	1.11 (0.88–1.38)	0.171
Endocrine glands	1 (Ref)	0.79 (0.29–2.13)	1.01 (0.63–1.63)	1 (Ref)	0.67 (0.27–1.69)	0.76 (0.49–1.16)	0.331

^a^ Adjusted for age, BMI, education level, smoking habits, alcohol consumption, activity at work, activity at home, diabetes, CVD, hypertension. ^b^ *p* < 0.05. ^c^ Interactions were calculated by inclusion of interaction terms. IR, incidence rate.

**Table 5:** Hazard ratios and 95% confidence intervals of mortality associated with mtDNA-CN (*n* = 491) and relative telomere length (*n* = 520) among cancer patients.

Characteristics	MtDNA-CN		HR (95% CI) per 1-SD Decrease in mtDNA-CN	Relative Telomere Length		HR (95% CI) per 1-SD Decrease in Telomere Length	*p* for Interaction ^c^
	High	Low		Long	Short		
**All cancer**							
No. of cancer patients	242	249		256	264		
No. of all-cause deaths	62	72		74	64		
No. of cancer-specific deaths	57	66		69	58		
Person-years of follow-up	1652	1578		1666	1787		
All-cause mortality rate, per 100 person-years	3.75	4.56		4.44	3.58		
Cancer specific mortality rate, per 100 person-years	3.45	4.18		4.14	3.25		
Adjusted all-cause mortality HR (95% CI)	1 (Ref)	1.14 (0.80–1.62)	1.20 (1.01–1.42) ^b^	1 (Ref)	0.78 (0.55–1.10)	0.86 (0.72–1.02)	0.090
Adjusted cancer-specific mortality HR (95% CI) ^a^	1 (Ref)	1.15 (0.80–1.66)	1.21 (1.01–1.45) ^b^	1 (Ref)	0.75 (0.53–1.07)	0.85 (0.71–1.00) ^b^	0.117
**Cancer types** (adjusted all-cause mortality HR and 95% CI) ^a^							
Breast cancer	1 (Ref)	1.42 (0.58–3.51)	1.16 (0.77–1.74)	1 (Ref)	0.61 (0.25–1.49)	0.73 (0.46–1.14)	0.952
Digestive system	1 (Ref)	1.08 (0.46–2.51)	1.53 (1.02–2.28)	1 (Ref)	1.36 (0.63–2.96)	0.86 (0.54–1.38)	0.959
Respiratory system	1 (Ref)	0.27 (0.06–1.25)	0.48 (0.18–1.24)	1 (Ref)	0.54 (0.14–2.20)	0.97 (0.50–1.89)	0.208
Genital organs	1 (Ref)	8.06 (1.75–37.2) ^b^	2.15 (1.04–4.44) ^b^	1 (Ref)	3.73 (0.86–16.2)	2.12 (1.00–4.52) ^b^	0.503
Urinary system	1 (Ref)	-	-	1 (Ref)	-	-	
Hematological cancer	1 (Ref)	0.05 (0.00–74.4)	-	1 (Ref)	0.24 (0.30–1.89)	-	
Nervous system	1 (Ref)	-	-	1 (Ref)	-	-	
Skin	1 (Ref)	-	-	1 (Ref)	-	-	
Endocrine glands	1 (Ref)	-	-	1 (Ref)	-	-	
**Cancer types** (adjusted cancer-specific mortality HR and 95% CI) ^a^							
Breast cancer	1 (Ref)	1.71 (0.67–4.35)	1.23 (0.86–1.77)	1 (Ref)	0.34 (0.11–1.04)	0.56 (0.34–0.95) ^b^	0.829
Digestive system	1 (Ref)	0.89 (0.34–2.39)	1.42 (0.90–2.25)	1 (Ref)	1.06 (0.46–2.44)	0.80 (0.48–1.34)	0.609
Respiratory system	1 (Ref)	0.34 (0.08–1.44)	0.48 (0.21–1.08)	1 (Ref)	0.83 (0.32–2.15)	1.19 (0.72–1.96)	0.421
Genital organs	1 (Ref)	5.59 (1.61–19.4) ^b^	2.42 (1.03–5.70) ^b^	1 (Ref)	4.56 (1.30–16.0)	1.98 (1.10–3.53) ^b^	0.167
Urinary system	1 (Ref)	-	-	1 (Ref)	-	-	
Hematological cancer	1 (Ref)	-	-	1 (Ref)	-	-	
Nervous system	1 (Ref)	-	-	1 (Ref)	-	-	
Skin	1 (Ref)	-	-	1 (Ref)	-	-	
Endocrine glands	1 (Ref)	-	-	1 (Ref)	-	-	

^a^ Adjusted for age, BMI, education level, smoking habits, alcohol consumption, activity at work, activity at home, diabetes, CVD, hypertension. ^b^ *p* < 0.05. ^c^ Interactions were calculated by inclusion of interaction terms. IR, incidence rate. -, not enough cases to conduct the analysis.

## Data Availability

The data that support the findings of this study are available from the Swedish National Board of Health and Welfare. Restrictions apply to the availability of these data, which were used under license for the current study, so supporting data are not publicly available.

## Supplementary Materials

The following are available online at https://www.mdpi.com/article/10.3390/cancers13153842/s1, Table S1: Spectrum of cancers in the WHILA study (1995–2015). Table S2: Baseline characteristics of cancer incidence. Table S3: Hazard ratios and 95% confidence intervals of cancer incidence and mortality associated with mtDNA-CN and relative telomere length stratified by smoking status, Figure S1: The coefficient (r) correlation between mtDNA-CN and age was −0.126 (*p* < 0.001), Figure S2: The coefficient (r) correlation between relative telomere length and age was −0.139 (*p* < 0.001), Figure S3: Kaplan-Meier plot for cancer mortality by mtDNA-CN categorized into two groups according to the median, Figure S4: Kaplan-Meier plot for cancer mortality by relative telomere length categorized into two groups according to the median.
