# Supplementary material for: Association of Mitochondrial DNA Copy Number and Telomere Length with Prevalent and Incident Cancer and Cancer Mortality in Women: A Prospective Swedish Population-Based Study
Source: Cancers (Basel). 2021 Jul 30;13(15):3842. doi: 10.3390/cancers13153842 (PMC8345403; doi:10.3390/cancers13153842)

Table S1. Spectrum of cancers in the WHILA study (1995-2015).

Characteristics	Prevalent cancers	Incident cancers	
	No. of cases (%)	No. of cases (%)	No. of fatal course (%)
**All Cancer**	187 (100)	520 (100)	138 (100)
**Breast Cancer**	77 (41.2)	186 (35.8)	23(16.7)
**Digestive system**			
Liver cancer	0	14 (2.7)	12 (8.7)
Pancreatic cancer	0	8 (1.5)	8 (5.8)
Gastric cancer	0	6 (1.2)	6 (4.3)
Small intestine cancer	0	4 (0.8)	0
Rectum cancer	1 (0.5)	9 (1.7)	4 (2.9)
Colon cancer	9 (4.8)	35 (6.7)	5 (3.6)
Oral cancer	1 (0.5)	7 (1.3)	1 (0.7)
**Respiratory system**			
Lung cancer	1 (0.5)	31 (6.0)	23 (16.7)
**Genital organ**			
Ovary cancer	14 (7.5)	20 (3.8)	16 (11.6)
Cervix cancer	5 (2.7)	0	0
Uterus cancer	1 (0.5)	0	0
Corpus cancer	15 (8.0)	29 (5.6)	4 (2.9)
**Urinary system**			
Kidney cancer	2 (1.1)	5 (1.0)	2 (1.5)
Urethral cancer	3 (1.6)	8 (1.5)	0
**Haematological cancer**			
Myeloma	0	3 (0.6)	2 (1.5)
Leukemia	3 (1.6)	15 (2.9)	7 (5.1)
Non-Hodgkin's lymphoma	4 (2.1)	24 (4.6)	4 (2.9)
**Nervous system cancer**	5 (2.7)	12 (2.3)	4 (2.9)
**Skin**			
skin	6 (3.2)	38 (7.3)	2 (1.5)
melanoma	12 (6.4)	28 (5.4)	2 (1.5)
**Endocrine glands cancer**			
Endocrine	7 (3.7)	16 (3.1)	1 (0.7)
Thyroid cancer	10 (5.3)	3 (0.6)	0
**Unknown primary**	2 (1.1)	10 (1.9)	9 (6.5)
**Other**	9 (4.8)	9 (1.7)	3 (2.2)

Table S2. Baseline characteristics of cancer incidence.

Characteristics	MtDNA-CN High (n=1406)	MtDNA-CN Low (n=1482)	*P* value[a]	Relative Telomere Length Long (n=1473)	Relative Telomere Length Short (n=1564)	*P* value[a]
Age, mean (SD)	56.85 (2.8)	57.46 (2.9)	<0.001	56.79 (2.8)	57.52 (2.9)	<0.001
BMI, mean (SD)	25.54 (3.9)	25.79 (4.3)	0.104	25.46 (4.1)	25.87 (4.2)	0.007
Education level, No. (%)			0.001			0.091
0-9	751 (53.4)	891 (60.1)		822 (55.8)	913 (58.4)	
10-11	220 (15.7)	194 (13.1)		198 (13.4)	226 (14.5)	
≥12	435 (30.9)	397 (26.8)		453 (30.8)	425 (27.2)	
Smoking habit, No. (%)			<0.001			0.059
Non-smokers	211 (15.0)	327 (22.1)		271 (18.4)	290 (18.5)	
Past smokers	18 (1.3)	33 (2.2)		18 (1.2)	37 (2.4)	
Current smokers	1177 (83.7)	1122 (75.7)		1184 (80.4)	1237 (79.1)	
Alcohol habit, No. (%)			0.014			0.066
No consumption	318 (22.6)	395 (26.8)		347 (23.6)	414 (26.5)	
<12 g/day	898 (63.9)	923 (62.4)		931 (63.2)	976 (62.4)	
≥12 g/day	190 (13.5)	164 (10.8)		195 (13.2)	174 (11.1)	
Activity at work, No. (%)			0.022			0.009
Low	436 (31.0)	391 (26.4)		453 (30.8)	412 (26.3)	
Moderate	642 (45.7)	714 (48.2)		693 (47)	748 (47.8)	
High	328 (23.3)	377 (25.4)		327 (22.2)	404 (25.8)	
Activity at home, No. (%)			0.353			0.04
Low	809 (57.5)	878 (59.2)		835 (56.7)	944 (60.4)	
High	597 (42.5)	604 (40.8)		638 (43.3)	620 (39.6)	
Comorbidity, No. (%)						
Diabetes	155 (11.0)	235 (15.9)	<0.001	193 (13.1)	223 (14.3)	0.355
CVD	231 (16.4)	293 (19.8)	0.020	252 (17.1)	295 (18.9)	0.209
Hypertension	570 (40.5)	630 (42.5)	0.283	614 (41.7)	652 (41.7)	0.998

CVD indicates cardiovascular disease. [a]Student t-tests-were performed for continuous variables. Chi-square tests were performed for categorical variables.

Table S3. Hazard ratios and 95% confidence intervals of cancer incidence and mortality associated with mtDNA-CN and relative telomere length stratified by smoking status.

Characteristics	MtDNA-CN		HR (95% CI) per 1-SD decrease in mtDNA-CN	Relative Telomere Length		HR (95% CI) per 1-SD decrease in telomere length	*P* for interaction [c]
	High	Low		Long	Short		
**Cancer incidence**							
**Current & past smokers**							
No. of individuals	229	360		289	327		
No. of cancer diagnosis	43	70		56	61		
Person-years of follow-up	3499	5254		4316	4820		
IR, per 1000 person-year	12.29	13.32		12.97	12.66		
Adjusted HR (95% CI) [a]	1 (Ref)	1.01 (0.69, 1.52)	0.96 (0.83, 1.10)	1 (Ref)	1.09 (0.75, 1.58)	1.08 (0.90, 1.30)	0.783
**Non-smokers**							
No. of individuals	1177	1122		1184	1237		
No. of cancer diagnosis	193	185		200	203		
Person-years of follow-up	18201	16984		18149	18735		
IR, per 1000 person-year	10.60	10.89		11.02	10.84		
Adjusted HR (95% CI) [a]	1 (Ref)	0.99 (0.81, 1.21)	1.00 (0.90, 1.11)	1 (Ref)	0.93 (0.77, 1.14)	0.98 (0.89, 1.08)	0.862
**Cancer mortality**							
**Current & past smokers**							
No. of cancer patients	44	69		56	61		
No. of all-cause death	9	27		21	35		
No. of cancer specific death	9	26		20	35		
Person-years of follow-up	387	555		467	507		
All-cause mortality rate, per 100 person-year	2.33	4.86		4.50	6.90		
Cancer specific mortality rate, per 100 person-year	2.33	4.68		4.28	6.90		
Adjusted all-cause mortality HR (95% CI)	1 (Ref)	2.63 (1.19, 5.83) [b]	2.01 (1.34, 3.21) [b]	1 (Ref)	0.68 (0.35, 1.33)	0.79 (0.56, 1.12)	0.027
Adjusted cancer specific mortality HR (95% CI) [a]	1 (Ref)	2.59 (1.17, 5.42) [b]	2.15 (1.35, 3.44) [b]	1 (Ref)	0.77 (0.37, 1.45)	0.80 (0.57, 1.12)	0.030
**Non-smokers**							
No. of cancer patients	198	180		200	203		
No. of all-cause death	53	45		53	48		
No. of cancer specific death	48	40		49	42		
Person-years of follow-up	1613	1530		1683	1643		
All-cause mortality rate, per 100 person-year	3.29	2.94		3.15	2.92		
Cancer specific mortality rate, per 100 person-year	2.96	2.61		2.91	2.56		
Adjusted all-cause mortality HR (95% CI)	1 (Ref)	0.94 (0.63, 1.42)	1.11 (0.91, 1.35)	1 (Ref)	0.86 (0.58, 1.29)	0.89 (0.73, 1.09)	0.243
Adjusted cancer specific mortality HR (95% CI) [a]	1 (Ref)	0.92 (0.60, 1.40)	1.08 (0.88, 1.32)	1 (Ref)	0.80 (0.53, 1.22)	0.86 (0.71, 1.05)	0.493

[a] Adjusted for age, BMI, education level, smoking habit, alcohol consumption, activity at work, activity at home, diabetes, CVD, hypertension. [b] $P < 0.05$. [c] Interactions were calculated by inclusion of interaction terms. IR, incidence rate.

Figure S1. The coefficient (r) correlation between mtDNA-CN and age was -0.126 (p< 0.001).

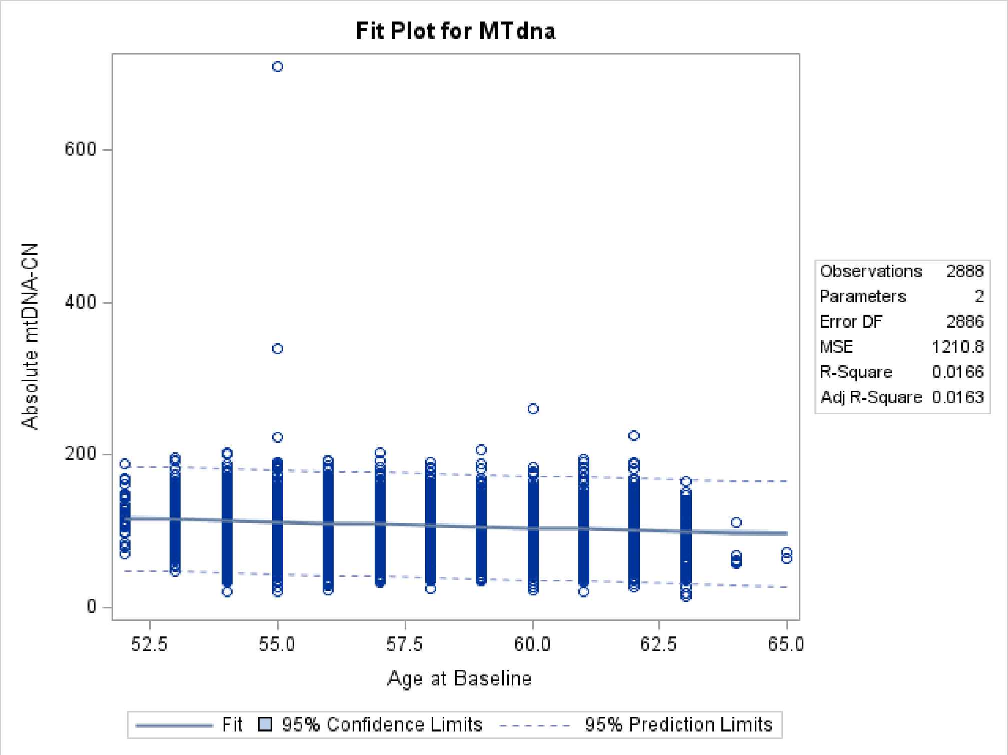

Figure S2. The coefficient (r) correlation between relative telomere length and age was -0.139 (p< 0.001).

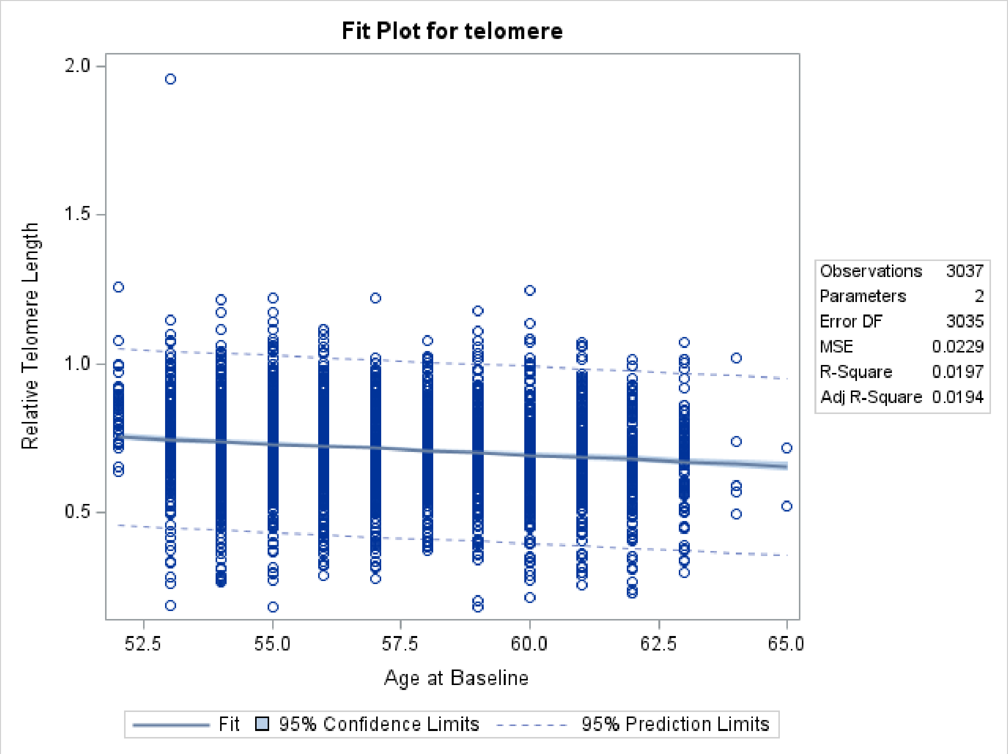

Figure S3. Kaplan-Meier plot for cancer mortality by mtDNA-CN categorized into two groups according to the median.

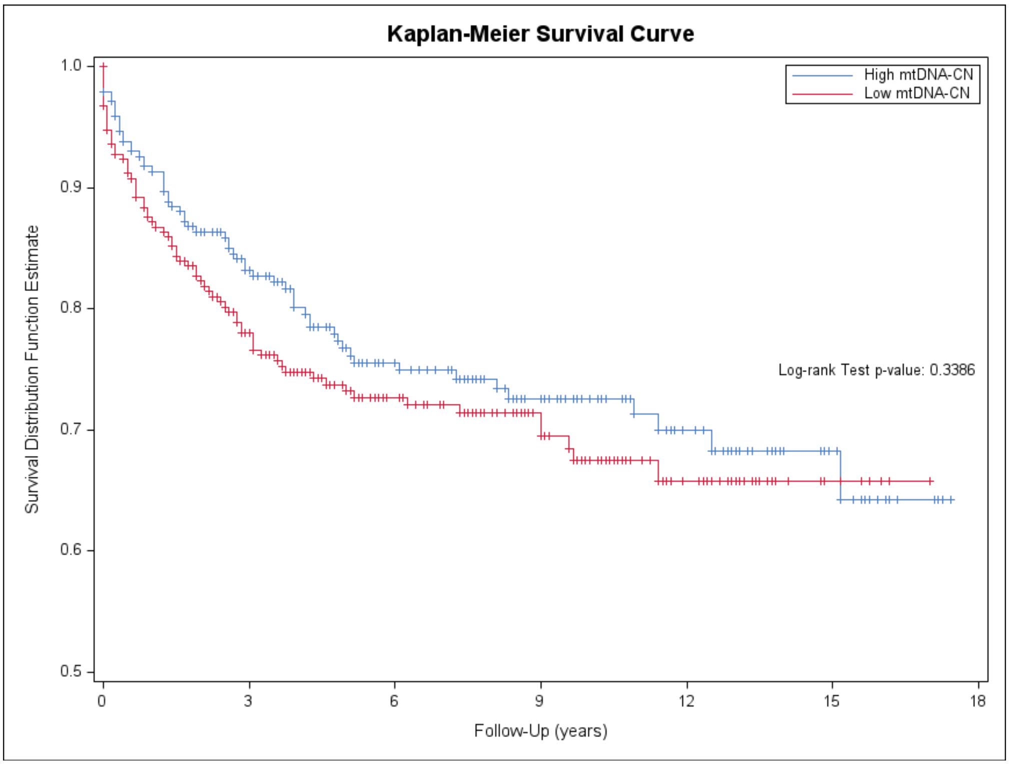

Figure S4. Kaplan-Meier plot for cancer mortality by relative telomere length categorized into two groups according to the median.

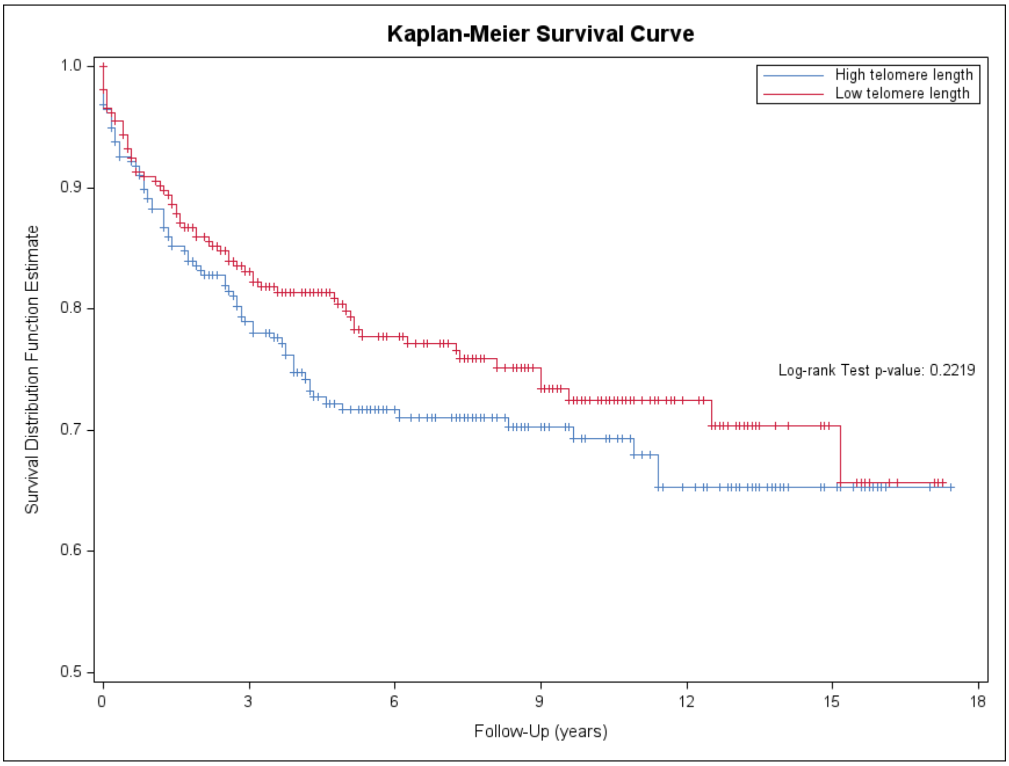

Supplement: Supplementary file 1 [file cancers-13-03842-s001.zip › cancers-1303032-supplementary.pdf]
